# High Concentrations of Atmospheric Ammonia Induce Alterations in the Hepatic Proteome of Broilers (*Gallus gallus*): An iTRAQ-Based Quantitative Proteomic Analysis

**DOI:** 10.1371/journal.pone.0123596

**Published:** 2015-04-22

**Authors:** Jize Zhang, Cong Li, Xiangfang Tang, Qingping Lu, Renna Sa, Hongfu Zhang

**Affiliations:** State Key Laboratory of Animal Nutrition, Institute of Animal Sciences, Chinese Academy of Agricultural Sciences, Beijing, China; Leibniz-Institut für Analytische Wissenschaften—ISAS Dortmund, GERMANY

## Abstract

With the development of the poultry industry, ammonia, as a main contaminant in the air, is causing increasing problems with broiler health. To date, most studies of ammonia toxicity have focused on the nervous system and the gastrointestinal tract in mammals. However, few detailed studies have been conducted on the hepatic response to ammonia toxicity in poultry. The molecular mechanisms that underlie these effects remain unclear. In the present study, our group applied isobaric tags for relative and absolute quantitation (iTRAQ)-based quantitative proteomic analysis to investigate changes in the protein profile change in hepatic tissue of broilers exposed to high concentrations of atmospheric ammonia, with the goal of characterizing the molecular mechanisms of chronic liver injury from exposure to high ambient levels of ammonia. Overall, 30 differentially expressed proteins that are involved in nutrient metabolism (energy, lipid, and amino acid), immune response, transcriptional and translational regulation, stress response, and detoxification were identified. In particular, two of these proteins, beta-1 galactosidase (GLB1) and a kinase (PRKA) anchor protein 8-like (AKAP8 L), were previously suggested to be potential biomarkers of chronic liver injury. In addition to the changes in the protein profile, serum parameters and histochemical analyses of hepatic tissue also showed extensive hepatic damage in ammonia-exposed broilers. Altogether, these findings suggest that longtime exposure to high concentrations of atmospheric ammonia can trigger chronic hepatic injury in broilers via different mechanisms, providing new information that can be used for intervention using nutritional strategies in the future.

## Introduction

Ammonia is a colorless, irritant gas generated from the nitrogenous fraction of animal wastes by microbial activity in the broiler house. With the development of the poultry industry, ammonia as a main contaminant in the air is causing increasing problems with broiler production. The greatest problem caused by ammonia in the air is reduced growth performance, which results in lower body weights and higher feed conversion ratios [1, 2]. Longtime exposure can create severe health issues and interfere with broiler welfare [3, 4].

Ammonia is a toxin in metabolic disorders associated with hyperammonemia, and pro-inflammatory mediators are released into the circulation, modulating the impact of ammonia on the brain tissue [5]. The neurotoxicity of ammonia induces increased expression of tumor necrosis factor α (TNF-α) and interleukin 1 β (IL-1β), which are associated with the production of reactive oxygen species (ROS) and nitric oxide (NO) that are involved with protein kinase A (PKA), the extracellular signal regulated kinase (ERK) pathway and nuclear factor-κB (NF-κB) activation in astrocytes in rats [6]. There is increasing evidence supporting the role of inflammation in exacerbating the neurological manifestations of both acute and chronic liver failure [7].

The liver is a central organ that plays a key role in diverse functions within the body. These functions include regulation of nutrient metabolism (carbohydrate, protein and lipid), immune response, inflammation and removal of xenobiotics [8, 9]. Hepatic function is also strongly influenced by stress and disease [10, 11]. A previous gene array study on broiler chickens demonstrated marked hepatic responses to aqueous ammonia-induced contact dermatitis, including genes associated with energy metabolism, thyroid hormone activity, cellular control and the pro-inflammatory response [3] In rat models of hyperammonemia-induced hepatic injury, microarray results demonstrated that altered expression of hepatic genes was relevant to many vital functions belonging to different signal transduction pathways [12]. To date, most studies of ammonia toxicity have focused on the nervous system and the gastrointestinal tract in mammals. However, few detailed studies have been conducted on the hepatic response to ammonia in poultry.

Numerous studies have demonstrated a lack of correlation between mRNA and protein abundance because of RNA editing and posttranslational modifications [13, 14]. Hence, the elucidation of protein expression is more accurate and imperative [15]. There are numerous enzymes in the liver that are involved in physiological functions related to nutrient metabolism, the immune response, and inflammation, among others [16]. It is almost impossible to detect all of the proteins in hepatic tissue at the same time using traditional methods, such as western blots, immunohistochemical staining or ELISAs. Currently, little is known about the alteration of proteins in the liver of poultry that have been exposed to high levels of atmospheric ammonia.

Based on previous research, we hypothesized that exposure to high concentrations of atmospheric ammonia can confer negative effects on the hepatic tissue of broilers via different mechanisms, which requires further study to elucidate. Therefore, in this study, we utilized a label-based iTRAQ procedure, followed by LC-MS/MS to quantitate altered proteins that are induced differentially in the livers of broilers exposed to high concentrations of atmospheric ammonia.

## Materials and Methods

### Ethics statement

This study was undertaken in strict accordance with the Regulations for the Administration of Affairs Concerning Experimental Animals of the State Council of the People’s Republic of China. The protocol was approved by the Committee on Experimental Animal Management of the Chinese Academy of Agricultural Sciences.

### Reagents and chemicals

Protein Assay Kit was purchased from Bio-Rad (Hercules, CA, USA). Reagent for total RNA isolation was from Qiagen (Valencia, CA, USA). Reagents for qPCR were obtained from (Takara, Dalian, China). All iTRAQ reagents and buffers were obtained from Applied Biosystems Inc. (ABI, Foster City, CA). The rest of reagent grade chemicals used in the present study were from Sigma Aldrich (St. Louis, MO, USA) or Fisher Scientific (Pittsburg, PA, USA).

### Animals and exposure conditions

Sixty one day old Arbor Acers male broilers were obtained from a commercial hatchery in Beijing (Beijing Arbor Acers Broiler Co., Beijing, China). All birds were housed in individual wire-bottom cages in an environmentally controlled room under standard brooding practices, and given *ad libitum* access to water and a maize-soybean basal diet during the first 21 days. Then, broilers were transferred to environmentally controlled exposure chambers. The diet during the experiment was formulated to achieve the National Research Council (NRC, 1994) recommended requirements for all nutrients containing ME, 12.76 MJ kg^-1^, and crude protein 19.94% (S1 Table). The concentrated ammonia was delivered in a whole-body animal exposure chamber [4] from days 22 to 42. Each exposure chamber was a 4500 × 3000 × 2500 mm (length × width × height) sealed unit, sectioned for housing 30 birds per chamber. Temperature and airflow were controlled during the exposures to ensure adequate ventilation, minimize buildup of animal-generated contaminants (dander, H_2_S, CO_2_) and to avoid thermal stress [17].

Broilers in the treatment group were exposed to 75 ± 3 μL/L ammonia during the experimental period. Broilers in the control group were raised in a separate chamber without ammonia for the same period, and the concentration of ambient ammonia was kept at 3 ± 3 μL/L. The concentration of ammonia in both chambers was monitored with a LumaSense Photoacoustic Field Gas-Monitor Innova-1412 (Santa Clara, CA, USA) during the entire experiment. Body weight (BW) and feed consumption were recorded weekly for performance evaluation.

### Sample collection

At day 42, all birds were weighed after a 12 h- fasting (12 h food withdrawal) period. Performance parameters (n = 30) including body weight gain, feed intake and feed-conversion ratio were determined. Twelve birds (6 per each group) were randomly selected for blood and hepatic sample collection. The procedure of all sample collections is previously described with slight modifications [12, 18]. Briefly, each blood sample was obtained from a wing vein using a sterilized syringe within 30 s. Blood was incubated in a water bath for 1 h at 37°C then centrifuged at 400 × g for 10 min at 4°C, and the sera obtained were stored at -80°C for further analysis [18]. After blood collection, the chickens were sacrificed by cervical dislocation and then exsanguinated. Then the abdominal and thoracic cavities were opened and hepatic tissues were collected. Small parts of hepatic tissues from both groups were fixed in 10% formaldehyde and used for histochemical analysis. The remaining hepatic samples were then washed with ice-cold sterilized saline, frozen in liquid nitrogen, and stored at -80°C for further proteome and qPCR analyses.

### Biochemical and histological analyses

For biochemical analysis, the activities of alanine aminotransferase (ALT), aspartate aminotransferase (AST), creatine kinase (CK) and total superoxide dismutase (T-SOD) in the serum were measured using a corresponding diagnostic kit (Nanjing Jiancheng Bioengineering Institute, Nanjing, China) according to the instructions of the manufacturer. For histological analysis, the protocol was described previously [18]. Fixed hepatic tissues in 10% phosphate-buffered formalin were processed for paraffin embedding, and 6 μm cross-sections were cut and placed on glass slides. The slides were dewaxed in xylene and rehydrated through gradient ethanol washes and then stained with hematoxylin and eosin (H&E) following standard procedures.

### Hepatic sample preparation and protein extraction

Sample pooling is a commonly used strategy to reduce the influence of individual variation on candidate target selection in proteomic studies [19–21]. To avoid erroneous conclusions due to individual variations, the same amount of hepatic tissue sample (weight: weight as 1: 1 ratio) from two chickens in the same group was pooled as a biological replicate, and three biological replicates were acquired for each group.

Hepatic protein extraction was performed as previously described with some modifications [22, 23]. Each pooled hepatic sample (~0.5 g) was ground in a Dounce glass grinder using liquid nitrogen. Ground samples were precipitated with 10% trichloroacetic acid (TCA) (w/v), 90% ice-cold acetone at -20°C for 2 h. The samples were then centrifuged at 20,000 × g for 30 min at 4°C. The supernatants were decanted and the pellets washed with ice-cold acetone. The pellets were lysed in lysis buffer consisting of 8 M urea, 30 mM 4-(2-hydroxyethyl)-1-piperazineethanesulfonic acid (HEPES), 1 mM phenylmethanesulfonyl fluoride (PMSF), 2 mM ethylene diamine tetraacetic acid (EDTA), and 10 mM dithiothreitol (DTT). The undissolved pellets in the crude tissue extracts were removed by centrifugation (20,000 × g, 30 min, 4°C). The tissue lysates were reduced for 1 h at 36°C in a water bath with a final concentration of 10 mM DTT by addition of 1 M DTT and then alkylated for 1 h in the dark with a final concentration of 55 mM by addition of 1 M iodoacetamide (IAM). After reduction and alkylation, 4 volumes of ice-cold acetone was added in the solution in order to precipitate proteins. Then proteins were washed three times with ice-cold pure acetone and resuspended in buffer containing 50% tetraethyl ammonium bromide (TEAB) and 0.1% sodium dodecyl sulfonate (SDS). The undissolved pellets were removed in protein samples by centrifugation (20,000 × g, 30 min, 4°C), and protein quantitation was determined by a Bio-Rad Bradford Protein Assay Kit (Hercules, CA, USA).

### Trypsin digestion

Each sample was digested overnight at 37°C adding sequencing grade trypsin (Promega Corporation, Madison, WI) at a 1: 30 ratio (3.3 μg trypsin: 100 μg target) [22].

### iTRAQ labeling

iTRAQ labeling procedure was described in previous report [22]. Protein samples in the control group were labeled with iTRAQ tags (solubilized in 70 μL isopropanol prior to use) 113, 114 and 115 while treatment samples received iTRAQ tags 116, 117, and 121. All labeled samples (organic composition > 60% by adding isopropanol) were incubated at room temperature for 2 h.

### Strong cation exchange chromatography

The strong cation exchange fractionation protocol followed previous reports [20, 23, 24] with slight modifications. Briefly, total amount of 600 μg sample was loaded onto a strong cation exchange column (Phenomenex Luna SCX 100A) equilibrated with buffer A (10 mM KH_2_PO_4_ in 25% acetonitrile, pH 3.0) using an Agilent 1100 (Santa Clara, CA) system. The peptides were separated using a linear gradient of buffer B (10 mM KH_2_PO_4_ and 2 M KCl in 25% acetonitrile, pH 3.0) increasing to 5% after 41 min, 50% after 66 min and 100% after 71 min, at a flow rate of 1 ml/min. Elution was monitored by measuring the absorbance at 214 nm. Total of 16 fractions were collected from the eluted peptides, and each fraction was desalted with a Strata X C18 column (Phenomenex) and vacuum-dried.

### Mass spectrometry

The protocol of MS analysis was described previously with slight modifications [20]. Each fraction was redissloved in buffer A (2% acetonitrile, 0.1% formic acid) and centrifuged at 20,000 × g for 10 min. The final concentration of peptides in each fraction was approximately 0.25 μg/μl on average. Twenty microliter of supernatant was loaded onto an UltiMate 3000 Nano LC system (Bannockburn, IL) by the auto sampler onto a C18 trap column (length 2 cm, inner diameter 200 μm). Peptides were eluted onto a resolving analytical C18 column (length 10 cm, inner diameter 75 μM, 5-μm particles, 300 Å) packed in-house. Samples were loaded at 15 μl/min for 4 min and eluted with a 45-min gradient at 400 nl/min from 5 to 60% buffer B (98% acetonitrile, 0.1% formic acid), separated with a 3- min linear gradient to 80% B, maintained at 80% B for 7 min, and finally returned to 5% B over 3 min. The peptides were subjected to nanoelectrospray ionization followed by tandem mass spectrometry (Q-Exactive, Thermo) coupled online to the nanoLC. Intact peptides were detected in the Orbitrap at a resolution of 70,000 full with width at half maximum (FWHM). Peptides were selected for MS/MS using high-energy collision dissociation (HCD) operating mode with a normalized collision energy setting of 28%; ion fragments were detected in the Orbitrap at 17,500 FWHM resolution. A data-dependent acquisition mode that alternated between a MS scan followed by MS/MS scans was applied for the 10 most abundant precursor ions (2^+^ to 4^+^) above a threshold ion count of 20,000 in the MS survey scan with a following Dynamic Exclusion duration of 15 s (isolation window of m/z 2.0 and a maximum ion injection time of 100 ms). The electrospray voltage applied was 1.8 kV. Automatic gain control (AGC) was used to optimize the spectra generated by the Orbitrap. The AGC target for full MS was 3E6 and 1E5 for MS2. For MS scans, the m/z scan range was 350 to 2000. For MS2 scans, the m/z scan range was 100–1800.

### Data processing and analyses

MS/MS data for iTRAQ protein identification and quantitation were analyzed using Proteome Discover 1.3 (Thermo Fisher Scientific) and searched with in-house MASCOT software (Matrix Science, London, U.K.; version 2.3.0) against the database Uniprot_Gallus gallus_9031 (Apr 11th, 2014 with 29,020 protein sequences) with the following parameters: enzyme: trypsin; fixed modification: carbamidomethyl (C); variable modifications: oxidation (M), gln-pyro-glu (N-term Q), iTRAQ 8-plex (N-term, K, Y); peptide mass tolerance:15 ppm; MS/MS tolerance: 20 mmu; maximum missed cleavages: 1. Identified peptides had an ion score above the threshold of peptide identity established by Mascot, and protein identifications were accepted within the false discovery rate (FDR) of 1% in which at least one such unique peptide match was specific for the protein. Median ratio normalization was performed in intra-sample channels in order to normalize each channel across all proteins. Protein quantitative ratios for each iTRAQ labeled sample were obtained, using a pooled sample in the control group (sample tagged with 113) as the denominator. Quantitative ratios were then log transformed to base 2 and presented as fold change relative to the denominator in the control group for final quantitative testing. Differentially expressed proteins were determined using Student’s *t*-test corrected for multiple testing performing the Benjamini and Hochberg correction [25]. Proteins with a 1.2-fold change or greater were considered to be differentially expressed. The mass spectrometry proteomics data have been deposited to the ProteomeXchange Consortium (http://proteomecentral.proteomexchange.org) via the PRIDE partner repository with the dataset identifier PXD001670.

### Bioinformatics analysis of proteins differential abundance

Gene Ontology (GO) distribution for all of the proteins that were significantly altered in hepatic samples of ammonia-exposed chickens were classified using Blast2GO software (http://www.blast2go.com/) and WEGO (http://wego.genomics.org.cn) that were provided by the Institute for Genomic Research [26, 27]. Database for Annotation, Visualization and Integrated Discovery (DAVID) 6.7 (http://david.abcc.ncifcrf.gov/) and the Kyoto encyclopedia of genes and genomes (KEGG) data base (released December 2014), were used to classify differentially expressed proteins in significantly overrepresented pathways and GO terms.

### Validation of proteins of differential abundance

Real-time quantitative PCR (qPCR) was used to verify eight hepatic tissue proteins of differential abundance at the mRNA level by the method previously described [18] with slight modifications. Total RNA from hepatic tissue samples was isolated using Qiagen RNeasy Plus Mini Kit (Valencia, CA). Agarose gel electrophoresis was used to test the RNA quality. And the RNA quantity was verified with a spectrophotometer (Nanodrop 2000, Thermo Scientific, Waltham, MA). Then total RNA was reverse transcribed using the Reverse Transcriptase, D2680A (Takara, Dalian, China). All mRNA expression levels were analyzed by RT-qPCR using the RT reagent Kit, RR037A (SYBR Green) (Takara, Dalian, China) and an Applied Biosystems 7500 Fast Real-Time PCR System (Foster City, CA). RT-qPCRs are performed as the following conditions: 95°C for 30 s, 40 cycles of 95°C for 10 s and 60°C for 30 s. Fluorescence data were detected at the last step of each cycle to monitor the amount of PCR product. The Primer sequences are shown in S2 Table. The relative fold-change was calculated by the 2^−ΔΔCT^ method [18].

### Statistical analysis

Data on growth performance, serum parameters and gene expressions were analyzed by one-way ANOVA (SAS Version 9.2, SAS institute Inc., Cary, NC). A group difference was assumed to be statistically significant when *P* < 0.05. All results were expressed as means ± S.D.

## Results

### Effect of high concentrations of atmospheric ammonia on the growth performance of broilers

In this study, treatment for all birds (control and treatment group) began on day 22. During the entire experimental period (20 days), birds exposed to ammonia had 15.4% less (*P* < 0.05) average daily gain (ADG) and 9.6% less (*P* < 0.05) average daily feed intake (ADFI). On the contrary, the feed conversion ratio (FCR) for the treatment group was greatly increased (*P* < 0.05) compared with the control group (Table 1).

**Table 1:** Effect of atmospheric ammonia on the growth performance of broilers.

	Groups	
	Control	Treatment
ADG (g/d)	91.08 ± 3.58^a^	77.03 ± 2.51^b^
ADFI (g/d)	149.45 ± 1.87^a^	135.14 ± 2.79^b^
FCR (g feed/g weight gain)	1.64 ± 0.09^b^	1.75 ± 0.05^a^

ADG, average daily gain; ADFI, average daily feed intake; FCR, feed conversion ratio. Values within a row not sharing a common superscript letter indicate significant difference at *P* < 0.05. Numbers are mean ± S.D. (n = 30).

### Effect of high concentration of atmospheric ammonia on the serum parameters and histochemistry of hepatic sample

**Fig 1:**
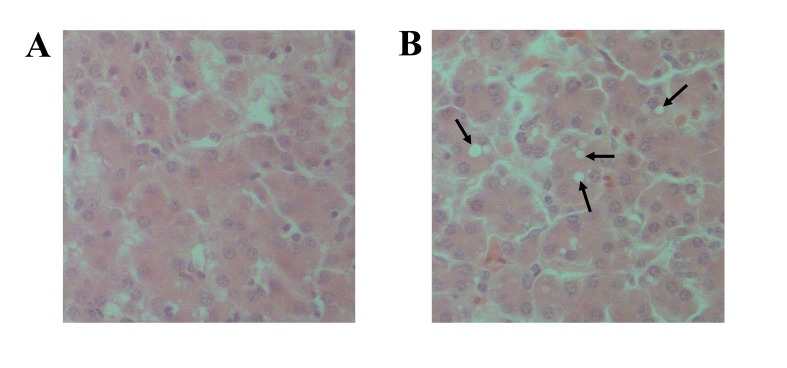
Histochemical examination of hepatic samples from broilers. Livers were fixed overnight in 10% phosphate-buffered formalin, and the tissue sections were prepared for hematoxylin and eosin staining. Representative images were captured by a microscope (Olympus BX51, Japan) at 400×; (A) CTRL, control group (3 ± 3 μL/L ammonia), (B) TRET, treatment group (75 ± 3 μL/L ammonia). In the TRET group, hepatocytes exhibited vacuole degeneration (arrow).

In the treatment group, activities of serum ALT, AST and CK were significantly elevated (*P* < 0.05) compared with the control group indicating extensive hepatic injury (Table 2). Antioxidase T-SOD and the serum level of ALB were decreased significantly compared with the control group (*P* < 0.05), illustrating lower oxidation resistance and synthetic capability (Table 2). For histological analysis, no obvious histological abnormalities were noted in the control group (Fig 1A). Compared with control broilers, the hepatocytes of ammonia-exposed broilers (Fig 1B) exhibited widespread vacuole degeneration.

**Table 2:** Effect of atmospheric ammonia on the serum biochemical parameters of broilers.

	Groups	
	Control	Treatment
ALT (IU/L)	19.33 ± 1.14^b^	24.87 ± 1.25^a^
AST (IU/L)	239 ± 5.18^b^	291 ± 5.65^a^
ALB (g/L)	14.75 ± 0.77^a^	11.45 ± 0.52^b^
CK (U/L)	6224.50 ± 172.26^b^	7173.63 ± 309.05^a^
T-SOD (U/mL)	77.81 ± 6.55^a^	61.12 ± 2.11^b^

ALT, activity of alanine aminotransferase; AST, activity of aspartate aminotransferase; ALB, albumin; CK, creatine kinase; T-SOD, total superoxide dismutase. Values within a column not sharing a common superscript letter indicate significant difference at *P* < 0.05. Numbers are mean ± S.D. (n = 6).

### Identification and comparison of proteins of differential abundance

Using iTRAQ analysis, a total of 79,527 peptide spectral matches were found, and 2452 proteins were identified within the FDR of 1% (S3 Table). Following statistical analysis, 34 proteins were found to be differentially expressed in hepatic tissue between the control and treatment groups, with 21 being up-regulated and 13 down-regulated (S4 Table).

**Fig 2:**
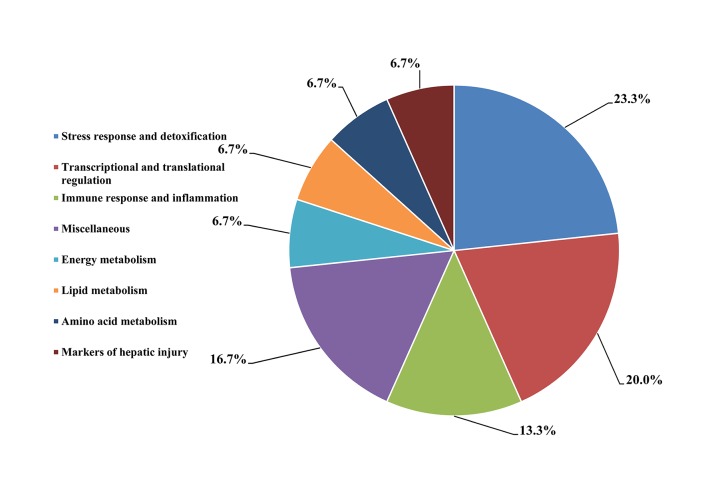
Functional classification of the proteins of differential abundance identified from the hepatic tissues of 42-day-old broilers.

A total of 30 proteins of differential abundance were grouped into eight classes based on putative functions: stress response and detoxification (23.3%), transcriptional and translational regulation (20.0%), immune response and inflammation (13.3%), miscellaneous (16.7%), energy metabolism (6.7%), lipid metabolism (6.7%), amino acid metabolism (6.7%) and markers of hepatic injury (6.7%) (Fig 2). Those related to stress response and detoxification, transcriptional and translational regulation, and immune response and inflammation were predominant and accounted for over 50% of the differentially- expressed proteins. A comparison of proteins of differential abundance with functional grouping between the two groups indicated that more protein species were up- regulated in ammonia-exposed chickens (18 versus 12, respectively) (Table 3). These 18 up-regulated protein species were distributed in six categories: five in stress response and detoxification, three in transcriptional and translational regulation, three in miscellaneous, two in lipid metabolism, two in amino acid metabolism and two in markers of hepatic injury (Table 3). The 12 down-regulated protein species were distributed in five categories: three in transcriptional and translational regulation, three in immune response and inflammation, two in stress response and detoxification, two in energy metabolism and two in miscellaneous. Most importantly, two protein species, beta-1 galactosidase (GLB1) and a kinase (PRKA) anchor protein 8-like (AKAP8 L), previously identified as potential biomarkers of hepatic injury, were found to be up-regulated in ammonia-exposed broilers in the present study.

**Table 3:** List of differentially expressed nutrients metabolic proteins in hepatic samples from treatment group and control group.

Accession	Description	Gene name	Log_2_ fold change	*P*-value	Biological process GO term
Energy metabolism					
Q5ZJ68	Uncharacterized protein OS = Gallus gallus GN = RCJMB04_20f2 PE = 2 SV = 1 - [Q5ZJ68_CHICK]	ACAD9	-0.29	0.0189	Acyl-CoA dehydrogenase activity
F1NSI8	Uncharacterized protein OS = Gallus gallus PE = 4 SV = 2 - [F1NSI8_CHICK]	None	-0.28	0.0420	GTP binding
Lipid metabolism					
E7EDS8	Fatty acid desaturase 1 OS = Gallus gallus GN = FADS1 PE = 2 SV = 1 - [E7EDS8_CHICK]	FADS1	0.31	0.0050	Fatty acid biosynthetic process
E1BYN1	Uncharacterized protein OS = Gallus gallus GN = LOC100858955 PE = 4 SV = 2 - [E1BYN1_CHICK]	LOC769339	0.42	0.0001	Fatty acid biosynthesis
Amino acid metabolism					
E1BZY3	Uncharacterized protein (Fragment) OS = Gallus gallus GN = A2LD1 PE = 4 SV = 2 - [E1BZY3_CHICK]	A2 LD1	0.35	0.0149	Cellular modified amino acid catabolic process
E1C9D0	Uncharacterized protein OS = Gallus gallus GN = DDO PE = 4 SV = 1 - [E1C9D0_CHICK]	DDO	0.39	0.0315	D-amino acid catabolic process
Transcriptional and translational regulation					
H9KZJ3	Uncharacterized protein OS = Gallus gallus PE = 4 SV = 2 - [H9KZJ3_CHICK]	None	-0.46	0.0242	Histone deacetylase activity
P09987	Histone H1 OS = Gallus gallus PE = 1 SV = 2 - [H1_CHICK]	None	-0.35	0.0284	DNA binding
F1NXK0	Mini-chromosome maintenance complex-binding protein OS = Gallus gallus GN = MCMBP PE = 4 SV = 2 - [F1NXK0_CHICK]	MCMBP	-0.32	0.0439	Chromatin binding
F1P4I9	Proteasomal ubiquitin receptor ADRM1 OS = Gallus gallus GN = ADRM1 PE = 2 SV = 1 - [F1P4I9_CHICK]	ADRM1	0.29	0.0299	Proteasome assembly
E1BUS2	Uncharacterized protein OS = Gallus gallus GN = NOP58 PE = 4 SV = 2 - [E1BUS2_CHICK]	NOP58	0.32	0.0425	snoRNA binding
F1N8A7	Uncharacterized protein OS = Gallus gallus GN = RBKS PE = 4 SV = 2 - [F1N8A7_CHICK]	RBKS	0.34	0.0182	Ribokinase activity
Immune response and inflammation					
E1BY93	Uncharacterized protein OS = Gallus gallus GN = IGJ PE = 4 SV = 1 - [E1BY93_CHICK]	IGJ	-0.38	0.0126	IgA binding
F1NIU6	Uncharacterized protein OS = Gallus gallus GN = M6PR PE = 2 SV = 2 - [F1NIU6_CHICK]	M6PR	-0.33	0.0362	Mannose transmembrane transporter activity
B8ZX71	Sixth complement component OS = Gallus gallus GN = C6 PE = 2 SV = 1 - [B8ZX71_CHICK]	C6	-0.28	0.0120	Innate immune response
F1NE09	Uncharacterized protein OS = Gallus gallus GN = CYFIP2 PE = 4 SV = 2 - [F1NE09_CHICK]	CYFIP2	0.29	0.0288	Protein binding
Stress response and detoxification					
H9KZK9	Uncharacterized protein OS = Gallus gallus PE = 4 SV = 2 - [H9KZK9_CHICK]	None	-0.50	0.0006	Scavenger receptor activity
F1P4G4	Uncharacterized protein OS = Gallus gallus GN = TBXAS1 PE = 3 SV = 2 - [F1P4G4_CHICK]	TBXAS1	-0.35	0.0101	Oxidoreductase activity
D0VX28	Cytochrome b-c1 complex subunit 6 OS = Gallus gallus PE = 1 SV = 1 - [D0VX28_CHICK]	UQCRH	0.31	0.0290	Mitochondrial electron transport
E1BUZ3	Uncharacterized protein OS = Gallus gallus GN = DHRS12 PE = 4 SV = 2 - [E1BUZ3_CHICK]	DHRS12	0.32	0.0079	Short-chain dehydrogenases activity
F1NMA3	Sulfotransferase family cytosolic 1B member 1 OS = Gallus gallus GN = SULT1B1 PE = 4 SV = 2 - [F1NMA3_CHICK]	SULT1B1	0.33	0.0336	Sulfotransferase activity
P08267	Ferritin heavy chain OS = Gallus gallus GN = FTH PE = 2 SV = 2 - [FRIH_CHICK]	FTH1	0.52	0.0068	Ferroxidase activity
E1C004	Uncharacterized protein (Fragment) OS = Gallus gallus GN = CA4 PE = 4 SV = 2 - [E1C004_CHICK]	CA4	0.64	0.0245	Carbonate dehydratase activity
Marker of hepatic injury					
Q5ZLM4	Uncharacterized protein OS = Gallus gallus GN = GLB1 PE = 2 SV = 1 - [Q5ZLM4_CHICK]	GLB1	0.26	0.0163	Beta-galactosidase activity
H9L081	Uncharacterized protein OS = Gallus gallus GN = AKAP8L PE = 4 SV = 2 - [H9L081_CHICK]	AKAP8 L	0.30	0.0444	DNA binding
Miscellaneous					
F1NDH2	Angiotensinogen OS = Gallus gallus GN = AGT PE = 3 SV = 2 - [F1NDH2_CHICK]	AGT	-0.37	0.0189	Acetyltransferase activator activity
F1NDD1	Uncharacterized protein (Fragment) OS = Gallus gallus GN = SFXN2 PE = 4 SV = 2 - [F1NDD1_CHICK]	SFXN2	-0.35	0.0102	Aation transmembrane transporter activity
F1NB83	Uncharacterized protein (Fragment) OS = Gallus gallus GN = TPP1 PE = 4 SV = 2 - [F1NB83_CHICK]	TPP1	0.32	0.0153	Serine-type endopeptidase activity
F1NQF6	Uncharacterized protein OS = Gallus gallus GN = MAN2B2 PE = 4 SV = 2 - [F1NQF6_CHICK]	MAN2B2	0.36	0.0212	Mannosidase activity
F1NVF2	Uncharacterized protein OS = Gallus gallus GN = MAP2K6 PE = 4 SV = 2 - [F1NVF2_CHICK]	MAP2K6	0.32	0.0105	MAP kinase kinase activity

A total of 30 proteins of differential abundance were grouped into eight classes based on putative functions. Protein expressions are represented as log_2_ fold change relative to the control

### GO annotations and pathway analysis

**Fig 3:**
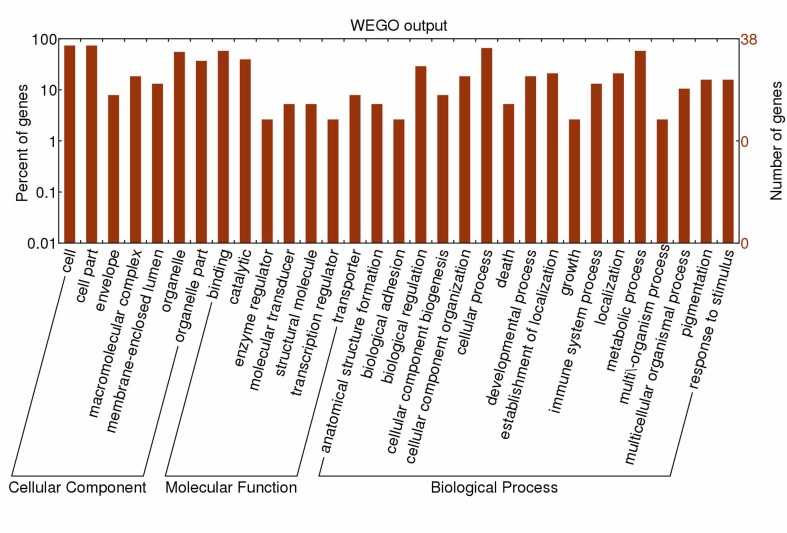
GO distribution analysis of differentially expressed proteins in hepatic tissues from treatment group and control group. The number of proteins for each GO annotation is shown in right axis, and the proportion of proteins for each GO annotation is exhibited in left axis.

In the cellular component group, the differentially expressed proteins are concentrated in intracellular organelles and the cytoplasm (Fig 3). In the molecular functional group, the differentially expressed proteins that are metabolic enzymes (oxidoreductase activity and hydrolase activity), binding proteins (protein binding and ion binding) and transporter (transmembrane), were ranked at the top of the category occupancy, suggesting that the relevant functions were important in the livers of broilers (Fig 3). In the biological process category, the proteins that participate in cellular processes (protein metabolic process), metabolism (protein and energy metabolism) and biological regulation (regulation of immune process), were at the top ratio in the differentially expressed proteins (Fig 3), suggesting that exposure to ammonia results mainly in changes in protein, energy metabolism and immune function.

In addition, GO annotation and KEGG pathway enrichment analysis were used to determine the overrepresented biological events and to provide a primary overview of the hepatic proteome influenced by exposure to ammonia. The DAVID 6.7 software identified that oxidation reduction in the biological process category is highly overrepresented (Table 4). The following proteins involved in this GO term were found to be enriched: TBXAS1, UQCRH, FADS1, ACAD9 and FTH1. These proteins might participate in the oxidation related metabolism. This functional enrichment analysis result indicated that exposure of ammonia had effect on oxidation system of chicken liver. KEGG pathway enrichment analysis of the differential proteins revealed information about protein functions in the metabolism pathway [21]. KEGG analysis showed that four differentially expressed proteins including MAN2B2, GLB1, TPP1 and M6PR, were significantly enriched in the two identified pathways. These proteins were involved in glycan degradation and lysosome (Table 4).

**Table 4:** Enriched KEGG pathway-based sets and GO terms of proteins of differential abundance in the hepatic tissue of broilers.

Category	Term	Count	Genes	*P* value
GO term	Oxidation reduction	5	TBXAS1, UQCRH, FADS1, ACAD9, FTH1	0.0042
KEGG pathway	Other glycan degradation	2	MAN2B2, GLB1	0.0489
KEGG pathway	Lysosome	3	TPP1, M6PR, GLB1	0.0499

ACAD9, acyl-CoA dehydrogenase family, member 9; FADS1, fatty acid desaturase 1; FTH1, ferritin heavy chain; GLB1, galactosidase, beta 1; MAN2B2, mannosidase, alpha, class 2B, member 2; M6PR, mannose-6-phosphate receptor; TBXAS1, thromboxane A synthase 1; TPP1, tripeptidyl peptidase I; UQCRH, ubiquinol-cytochrome C reductase hinge protein; The number of count refers to the amount of proteins involved in the extended KEGG network and pathway. *P* values are calculated according to a modified Fisher’s exact test and corrected for multiple testing using the Bonferroni correction provided by DAVID.

### Validation of proteins of differential abundance

**Fig 4:**
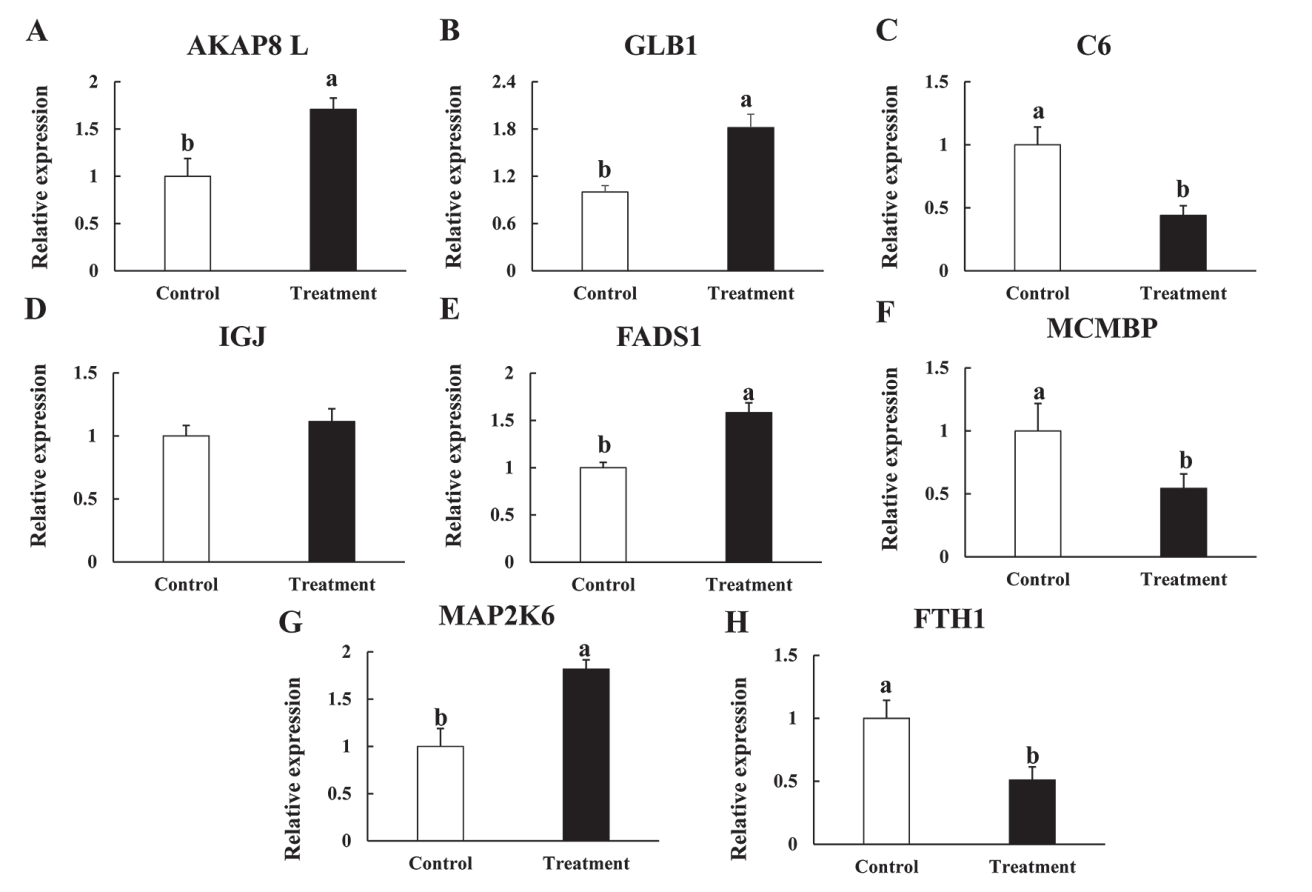
qPCR validation of eight proteins of differential abundance from the hepatic tissues of 42-day-old AA broilers at the mRNA level (A, B, C, D, E, F, G and H). Samples were normalized with the reference gene β-actin. Data are presented as means ± S.D (n = 6 per group). Mean values with different superscript letters (^a, b^) are significantly different (*P* < 0.05).

Eight differentially expressed proteins (AKAP8 L and GLB1 as potential biomarkers of hepatic injury, C6 and IGJ involved in immune response, FADS1 involved in lipid metabolism, MCMBP involved in transcriptional and translational regulation, MAP2K6 involved in receptor signaling, and FTH1 involved in stress response) were selected for validation of proteomic data at the mRNA level using qPCR (Fig 4). The protein levels of AKAP8 L, GLB1, C6, FADS1, MCMBP and MAP2K6 were consistent with their mRNA expression levels. The results for the remaining two proteins were inconsistent between the mRNA levels and the protein levels. Possible reasons for these inconsistent results include the following: 1) the relationships between the mRNA levels and the protein levels were indirect that some proteins were encoded by more than one gene, 2) there were some post-translational effects and/or the function of other regulatory mechanisms such as ubiquitin, proteolysis, regulation of microRNA and so on [13, 14], and 3) there was a time delay between responses on the mRNA and protein levels [21].

## Discussion

The liver is an important organ for nutrient metabolism and detoxification. Any damage to hepatocytes can trigger severe healthy issues in the body. Whether an exposure to high concentrations of ammonia has a direct effect on the functional hepatocytes, thereby causing synthetic and metabolic disorder of broiler’s liver, remains unknown. Much less is understood regarding the molecular mechanism of hepatic injury in poultry induced by exposure to atmospheric ammonia. In the present study, iTRAQ-based quantitative proteomic analysis integrated with biochemical detections was performed to investigate the hepatic response in ammonia-exposed broilers.

Serum levels of ALT, AST and CK serve as important indicators of hepatic function and were found to be significantly higher in ammonia-exposed broilers, indicating that ammonia exposure severely impaired hepatic function. In addition, lower levels of T-SOD and ALB in the serum of the treatment group suggested that the hepatic injury was associated with oxidative stress and disorders of synthetic functions. The result of histochemical analysis was consistent with changes of serum parameters and showed that a proportion of hepatocytes began to display widespread vacuole degeneration in ammonia-exposed broilers.

As an essential metabolic organ, the liver plays a vital rule in the production of ATP, which is involved in a number of physiological processes. However, ATP production is usually found to be impaired in animal models of hepatic injury. In hepatic cirrhosis patients, although mitochondrial function and ATP generation were maintained by increasing energy production from glycolytic flux at an early phase, mitochondria respiration and ATP production were significantly compromised at the terminal stage of hepatic injury [28]. Chronic administration of toxins that induce hepatic damage in a mouse model demonstrated that liver ATP-content was reduced to 58% of controls after 2 weeks of intoxication [29]. Both decreased glycolytic and mitochondrial ATP production were also demonstrated in a chronic alcoholic liver injury model [30]. These results are consistent with our discovery that abundances of the proteins encoded by acyl-CoA dehydrogenase gene (ACAD9) and a GTP binding protein (Uniport database accession F1NSI8) related to energy production [31] were down-regulated in hepatic samples of ammonia- exposed broilers. GO annotation enrichment analysis of the present study also showed that oxidation reduction term is overrepresented in hepatic proteome of ammonia- exposed broilers. Additional determinations are still required on physiological parameters such as ATP content, oxidative phosphorylation and so on in the future.

FADS1 participates in hepatic lipogenesis and catalyzes the conversion of long chain saturated fatty acids to monounsaturated fatty acids [32, 33]. The protein encoded by the fatty acyl-CoA hydrolase precursor gene (LOC769339) has been investigated previously, and its induction represents an adaptive response to fatty acid overload in the liver of animals fed a high-fat diet [34]. A previous study demonstrated that accumulation of fat in hepatocytes was shown in broilers raised under higher concentration of ammonia [35]. Up-regulation of these two proteins was observed in livers of ammonia- exposed birds and is consistent with the previous research. However, direct evidence is still required, such as fat content and profile of fatty acids, which will be involved in the additional study in the future.

Amino acid metabolism is one of the key functions in the liver and is an important indicator of the health status of hepatocytes. A2 LD1 (AIG2-like domain 1) is involved in proteolytic degradation, which is considered to be a biomarker in amniotic fluid during gestosis [36]. DDO (D-aspartate oxidase) acts as a detoxifying agent to metabolize D-amino acids [37]. Up-regulation of these two proteins indicates that exposure to ammonia may trigger perturbations in amino acid metabolism in hepatocytes. The determination of hepatic profile of amino acids will be considered in further study.

Transcriptional and translational regulatory proteins have crucial roles in the growth, proliferation and differentiation of hepatocytes. In this study, six differentially expressed protein species related to transcriptional and translational regulation were up- and down-regulated in the treatment group, respectively. Histone H1 and a histone deacetylase protein (Uniport database accession H9KZJ3) are involved in the regulation of gene expression, and down-regulation of both proteins was reported to be associated with impairment of liver regeneration [38]. MCMBP is known to be crucial for DNA replication, regulating initiation and elongation of DNA and providing DNA helicase activity [39]. ADRM1 is an integral plasma membrane protein that promotes cell adhesion, which has been shown to be induced in hepatocellular carcinoma (HCC) [40]. NOP58 (NOP58 ribonucleoprotein) is essential for ribosomal biogenesis, whereas an increased expression of this protein was observed in hyperglycemia-induced vascular injury [41, 42]. RBSK (ribokinase) is an important component of the first step of ribose metabolism; however, its elevated expression was demonstrated in livers of patients having pancreatic cancer metastasis [43]. Down-regulation of histones and MCMBP, together with up-regulation of ADRM1, NOP58 and RBSK observed in the present study, indicates that proliferation of normal hepatocytes was restrained, whereas the number of aberrant hepatocytes was increased.

In addition to having roles in nutrient metabolism, the liver is also an important organ for immunity and detoxification *in vivo*. In this study, 11 differentially-expressed proteins were identified in the categories of immune response, stress response and detoxification. Of these proteins, IGJ is active during the early stages of both B and T cell differentiation [44]; M6PR (mannose-6-phosphate receptor) plays a critical role in lysosome function through the specific transport of mannose-6-phosphate-containing acid hydrolases from the Golgi complex to lysosomes [45]; C6 reduces risks for disseminated Neisserial infections [46]. Down-regulation of immune and anti-inflammatory response-related proteins in this study indicates that the immunity of ammonia-exposed broilers was diminished, which renders broilers susceptible to bacterial or viral infections. However, up-regulation of apoptosis related CYFIP2 (cytoplasmic FMR1-interacting protein 2) may trigger the death of hepatocytes in the treatment group [47]. Furthermore, protein species related to oxidative stress and detoxification were up- and down-regulated in ammonia-exposed broilers, respectively. Elevated abundances of protein related to oxidative stress, including UQCRH, SULT1B1, CA4 (carbonic anhydrase 4), DHRS12 (dehydrogenase/reductase (SDR family) member 12) and FTH1 were observed in the present study. Up-regulation of these proteins is closely related to the formation of reactive oxygen species [48–51]. Together with the result of decreased serum level of T-SOD in the treatment group, we conclude that the exposure to ammonia induces oxidative stress in hepatocytes. This behavior is consistent with the increased oxidative stress in astrocytes induced by the neurotoxicity of ammonia [6]. In contrast, down-regulation of a scavenger receptor protein (Uniport database accession H9KZK9) involved in detoxification was demonstrated in the treatment group [52]. We speculate that the ability to remove xenobiotics may be impaired in hepatocytes of ammonia-exposed broilers.

Notably, two proteins previously identified as potential biomarkers of hepatic injury were found to be differentially expressed in the present study. GLB1 is a member of a group of enzymes that are contained predominantly within lysosomes and are released during Kupffer cell activation or death. It has been proposed as a marker of liver injury induced by radiation [53]. A rapid elevation of GLB levels corresponded well with impaired liver function and extensive hepatic injury [54]. AKAP8 L is involved in nuclear envelope breakdown and chromatin condensation. In high glucose induced diabetic liver disease, AKAP8 L was regarded as a potential marker of diabetic liver diseases [55]. Both proteins were greatly up-regulated in livers of ammonia-exposed broilers. Thus, we suggest that GLB1 and AKAP8 L are potential biomarkers of ammonia induced chronic hepatic injury in broilers.

In addition to the aforementioned protein clusters, differential abundances of several proteins involved in a broad array of functions were observed in this study. Of these proteins, MAP2K6 (mitogen-activated protein kinase kinase 6) plays a crucial role in the p38 MAP kinase signal cascade that regulates various stress-induced responses under pathological conditions [17, 56]. Previous research has demonstrated that the stress-activated MAPK cascade and p38 were main factors in chronic liver injury in hepatocarcinogenesis [57, 58]. An increased abundance of MAP2K6 was observed in our study, which indicates that the mechanism of ammonia-related liver injury may be related to the MAP kinase (MAPK) signaling pathway. However, this speculation is only derived from the present study and requires a huge effort to prove in further research. As similar as GLB1, TPP1 (tripeptidyl peptidase I) is also an important lysosomal protease [59]. KEGG pathway analysis in the present study showed that both proteins are involved in lysosome. This observation indicates that lysosome may play a vital role in hepatocytes of ammonia-exposed broilers.

In summary, the present proteomic analysis has demonstrated that exposure to high concentrations of atmospheric ammonia leads to differential abundances of a number of hepatic proteins in broilers. The functional groupings of those altered proteins are mainly related to stress response, immunity, and transcriptional and translational regulation. Some other proteins involved in nutrient metabolism (energy, lipid and amino acid), and receptor signaling were also differentially expressed. Most importantly, two proteins (GLB1 and AKAP8 L) previously identified as potential biomarkers of hepatic injury, were found in ammonia-exposed broilers. Altogether, the results of proteomic, biochemical and histological analyses, suggest that exposure to high concentrations of atmospheric ammonia triggers severe chronic hepatic injury through oxidative stress and some other ways in broilers. Future studies will investigate the regulatory mechanisms responsible for attenuating damages induced by atmospheric ammonia.

## Supporting Information

S1 Table Composition of the experimental diet and calculated proximate composition of the diet. (DOC)

S2 Table The qPCR primers used for verification of the differentially expressed genes of the AA broiler hepatic tissues. (DOC)

S3 Table List of all proteins (n = 2452) identified in the study. (XLS)

S4 Table List of all differentially expressed proteins (n = 34) identified in the study. (XLS)
