# Supplementary material for: High Concentrations of Atmospheric Ammonia Induce Alterations in the Hepatic Proteome of Broilers (Gallus gallus): An iTRAQ-Based Quantitative Proteomic Analysis
Source: PLoS One. 2015 Apr 22;10(4):e0123596. doi: 10.1371/journal.pone.0123596 (PMC4406733; doi:10.1371/journal.pone.0123596)
Supplement: S1 Table — (DOC) [file pone.0123596.s001.doc]

**Table S1. Composition of the experimental diet and calculated proximate composition of the diet.**

| **Ingredients (%)** | |
| --- | --- |
| Maize | 59 |
| Soybean meal | 32.4 |
| Soybean oil | 4 |
| Limestone | 1.15 |
| Calcium hydrophosphate | 1.65 |
| Lysine | 0.18 |
| Methionine | 0.33 |
| Choline chloride (50%) | 0.05 |
| Sodium chloride | 0.25 |
| Premix^1^ | 1 |
| Total | 100 |
| **Calculated nutrient and energy levels** |  |
| Crude protein (%) | 19.94 |
| Calcium (%) | 0.09 |
| Available P (%) | 0.40 |
| Lysine (%) | 1.14 |
| Methionine (%) | 0.50 |
| ME (MJ/kg) | 3.05 |

^1^Providing the following (g/kg fresh weight), Vitamin A, 5,000 IU; Vitamin D: 10,000 IU; Vitamin E: 75.0 mg; Vitamin K_3_, 18.8 mg; Vitamin B_1_, 9.8 mg; Vitamin B_2_, 28.8 mg; Vitamin B_6_, 19.6 mg; Vitamin B_12_, 0.1 mg; calcium pantothenate, 58.8 mg; nicotinic acid, 196.0 mg; folic acid, 4.9 mg; biotin, 2.5 mg; Cu (copper sulfate), 4.0 mg; Fe (ferrous sulfate), 40.0 mg; Zn (zinc sulfate), 37.6 mg; Mn (manganese sulfate), 50.0 mg; Se (sodium selenite), 0.2 mg; I (potassium iodide), 0.2 mg.
