# Supplementary material for: High Concentrations of Atmospheric Ammonia Induce Alterations in the Hepatic Proteome of Broilers (Gallus gallus): An iTRAQ-Based Quantitative Proteomic Analysis
Source: PLoS One. 2015 Apr 22;10(4):e0123596. doi: 10.1371/journal.pone.0123596 (PMC4406733; doi:10.1371/journal.pone.0123596)
Supplement: S2 Table — (DOC) [file pone.0123596.s002.doc]

**Table S2.** The qPCR primers used for verification of the differentially expressed genes of the AA broiler hepatic tissues.

| **Gene name** | **Primer sequence** | **Product length (bp)** |
| --- | --- | --- |
| β-actin | forward: GAGAAATTGTGCGTGACATCA  reverse: CCTGAACCTCTCATTGCCA | 152 |
| FTH1 | forward: GCTGCACAAATTGGCAACTG  reverse: CCATCTTCCGCAGGTTGGTC | 128 |
| C6 | forward: GAACTCGGGTCTGTCAGTGG  reverse: GACCGTTCTGCTGACTCCAA | 475 |
| IGJ | forward: ATTGTCCCACTCAAGAGCCG  reverse: AGGTGTAGCAGGTTTCTGGC | 181 |
| GLB1 | forward: GCACAGAGCCAACACAACTG  reverse: GGCAAGTCTGGAATCCCTCC | 406 |
| AKAP8 L | forward: GGACCTCACAGGGAAGAAGC  reverse: CTTGAGTTTTCTTGCCCGCC | 263 |
| MAP2K6 | forward: TACCTCGTTGACTCGGTTGC  reverse: TCTGGGTATGTTGGCCGTTC | 314 |
| FADS1 | forward: AGGGTATCCTGGGGCTTCAT  reverse: CTTTCAAAGAGTGGTGCTCG | 219 |
| MCMBP | forward: TCGTTCCAGCATCCTATCGC  reverse: TCGGCCCTCTGAGGTGATAA | 299 |

FTH1 = ferritin heavy chain; C6 = sixth complement component; IGJ = immunoglobulin J polypeptide; GLB1 = beta-1 galactosidase; AKAP8 L = a kinase (PRKA) anchor protein 8-like; MAP2K6 = mitogen-activated protein kinase kinase 6; FADS1 = fatty acid desaturase 1; MCMBP = mini-chromosome maintenance complex-binding protein.
